# Endoscopic Treatment of Bouveret Syndrome with Combined Laser and Mechanical Lithotripsy: A Case Report

**DOI:** 10.3390/jcm14051530

**Published:** 2025-02-25

**Authors:** Stefanie Parisi, Dario D’Agostino, Concetta Elisabetta Di Bartolo, Carlo Petruzzellis, Alessandra Scamporrino, Salvatore Piro, Domenico Catarella

**Affiliations:** 1 Gastroenterology Unit, Garibaldi-Nesima Hospital, 95122 Catania, Italy; stparisi@arnasgaribaldi.it (S.P.); ddagostino@arnasgaribaldi.it (D.D.); cdibartolo@arnasgaribaldi.it (C.E.D.B.); cpetruzzellis@arnasgaribaldi.it (C.P.); dcatarella@arnasgaribaldi.it (D.C.); 2 Department of Clinical and Experimental Medicine, Internal Medicine, Garibaldi-Nesima Hospital, University of Catania, 95122 Catania, Italy; alessandraska@hotmail.com

**Keywords:** Bouveret syndrome, endoscopy, lithotripsy

## Abstract

**Background:** Bouveret syndrome is a complication of cholelithiasis, characterized by the migration of a large gallstone from the gallbladder to a part of the stomach or intestine through a bilio-enteric fistula. This condition results in a rare form of gallstone ileus, presenting signs and symptoms of gastric outlet obstruction. **Methods:** This case report aims to present a rare instance of Bouveret syndrome in a 64-year-old woman who presented to our emergency department with recurrent epigastric pain and vomiting for over 2 months. After a CT scan, an esophagogastroscopy was performed following a multidisciplinary discussion. An endoscopic evaluation revealed a large (4 cm) gallstone found in the proximal duodenum using an endoscope. We then inserted the holmium laser fiber system through a standard ERCP catheter, passing it through the endoscope’s working channel. By positioning the holmium laser fiber within the catheter, we stabilized the energy on the gallstone, which was then fragmented into smaller pieces after administering pulse energy. **Results:** In this case report, we successfully treated Bouveret syndrome using endoscopic laser lithotripsy combined with mechanical lithotripsy, avoiding traditional surgery. **Conclusions:** The endoscopic approach that combines laser and mechanical lithotripsy appears effective in fragmenting large gallstones into smaller pieces, facilitating their passage through the digestive tract and resolving the obstruction.

## 1. Introduction

Bouveret syndrome is characterized by the migration of a large gallstone from the gallbladder to a portion of the stomach or intestine through a bilio-enteric fistula. This syndrome is a rare form of gallstone ileus, mainly caused by the obstruction at the gastric outlet and occasionally in other portions of the intestine [1].

Only about 315 cases have been reported in the literature in the past 50 years, from 1967 to 2016 [2], and it is indeed a rare complication of cholelithiasis cases (0.3–0.5%). This condition typically occurs in older women with a non-specific clinical presentation that is not limited to gastric outlet obstruction [3].

The rarity of Bouveret syndrome, its clinical complexity, and the absence of standardized guidelines limit the management of this condition, including endoscopic and surgery [4].

The treatments for Bouveret syndrome described in the literature are complex and varied. The choice of treatment depends on many factors, such as the patient’s age and comorbidities, the location of the obstruction, and the level of expertise.

The most widely used mechanisms in clinical practice include endoscopic removal with baskets and snares, but above all, endoscopic lithotripsy: mechanical lithotripsy, electrohydraulic lithotripsy, and laser lithotripsy; these new innovative techniques have allowed an improvement in the therapeutic success rate in recent years [2].

Here, we describe a case of Bouveret syndrome recently managed by our group. This case was treated through an endoscopic approach, which was successful, and the patient was discharged to home without complications.

## 2. Case Description

A 64-year-old woman presented to our emergency department with recurrent epigastric pain and vomiting for more than 2 months.

An endoscopic evaluation performed at another center the day before admission described a large gallstone impacted in the proximal duodenum.

The patient’s past medical history included hypertension and hypertrophic cardiomyopathy.

At the admission, the patient presented neutrophilic leukocytosis and dehydration, and as the reported symptoms persisted, it was decided to perform a nasogastric decompression with the placement of a nasogastric tube in the stomach, electrolyte replacement, and intravenous antibiotics were administered. We conducted an emergency CT scan to assess the overall morphological condition. Only an intravenous contrast medium was used for the abdominal CT scan; no oral contrast was necessary. Endoscopic ultrasound (EUS) was unnecessary due to the urgent clinical situation and the fact that the fistula was already visible on the contrast-enhanced CT scan. The gallbladder showed no stones, but there was significant wall inflammation.

**Figure 1:**
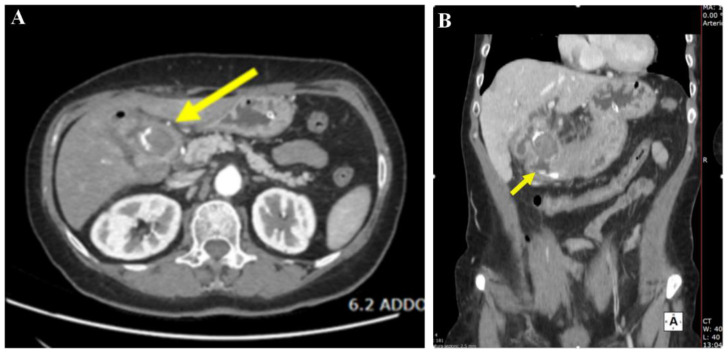
Computed tomography (CT). In panel (**A**) (axial section) and panel (**B**) (coronal section), a large 37 mm gallstone is shown obstructing the duodenal bulb (yellow arrow), resulting in upstream dilation of the gastric lumen.

The Computerized tomography (CT) of the abdomen with intravenous contrast media revealed a gallstone of 37 mm impacted at the gastro-duodenal passage, secondary to a gallbladder fistula. CT also showed aerobilia at the level of the intrahepatic bile ducts, depleted gallbladder, and distended gastric lumen (Figure 1).

**Figure 2:**
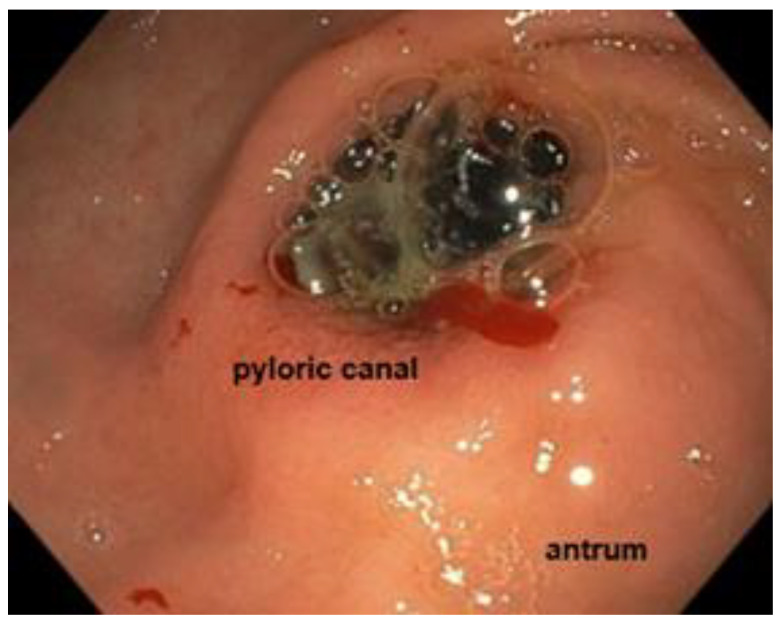
Endoscopic view showing the impacted gallstone in the proximal duodenum.

An esophagogastroscopy was performed the day after admission after a multidisciplinary discussion with anesthesiologists, radiologists, and surgeon colleagues. Endoscopic evaluation was performed with a GF H190 endoscope (Olympus, Tokyo, Japan). The patient was on orotracheal intubation in the supine decubitus position. The large 4 cm gallstone was impacted in the proximal duodenum (Figure 2).

**Figure 3:**
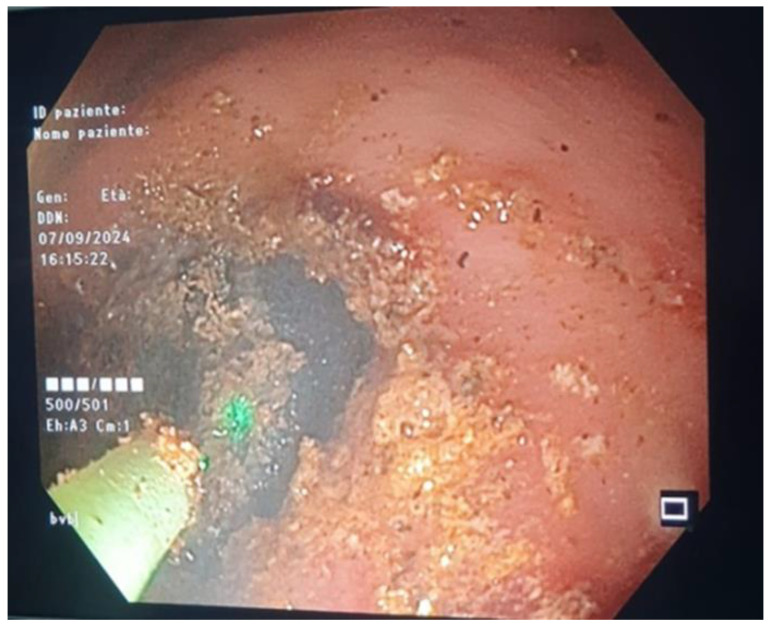
Endoscopic view showing the administration of a Holmium laser strike to the gallstone.

After many repeated attempts with a 25 mm diameter snare, we did not succeed in bringing the stone back into the stomach. Then, after a short consultation with our urologists, we inserted the holmium laser fiber namely Lumenis Pulse™ 100H Holmium Laser System (Boston Scientific S.p.A., Milan, Italy) through a standard ERCP catheter (diameter 1.8 mm), which was passed through the working channel of the endoscope by inserting the holmium laser fiber into the catheter, we were able to stabilize the energy on the gallstone, then the stone was broken into two pieces after the administration of pulse energy [2 J pulse energy and 30 W of power at the fiber tip, with a frequency 15 Hz] (Figure 3).

Subsequently, we performed mechanical lithotripsy to split the stone into small fragments of 2 cm. The following attempts at endoscopic removal of the fragments were difficult, probably due to the hyper-angulation of the duodenum deformed by the large fistula. As a result, we interrupted the procedure, which lasted about 3 h.

The procedure was uncomplicated, and no immediate adverse events were encountered. The patient had complete resolution of the symptoms after the endoscopic procedure.

**Figure 4:**
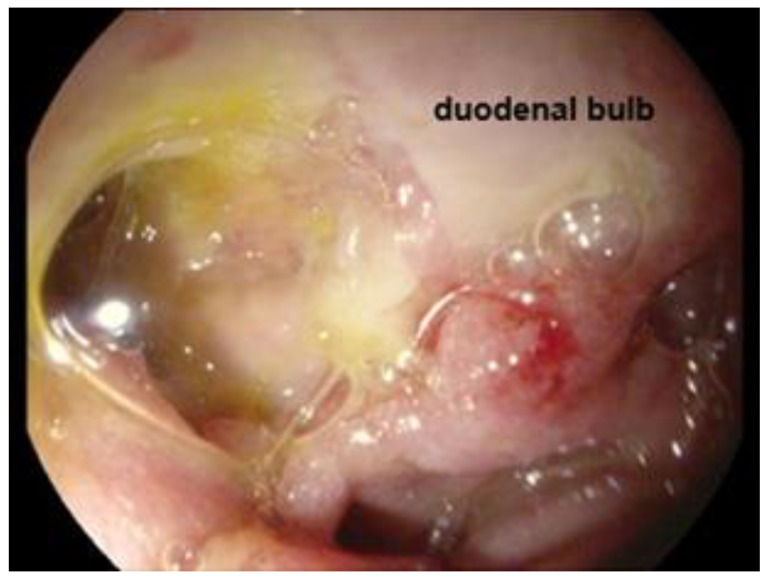
Endoscopy after 48 h showing a large fistula and a clean-based ulcer in the duodenal bulb.

We repeated endoscopy after 48 h with an EG-2990K (Pentax, Tokyo, Japan), then we described the large fistula in the duodenal bulb, diameter 3 × 4 cm, and adjacent to it a clean-based ulcer of 6 mm. No gallstones were visualized in the duodenum (Figure 4).

**Figure 5:**
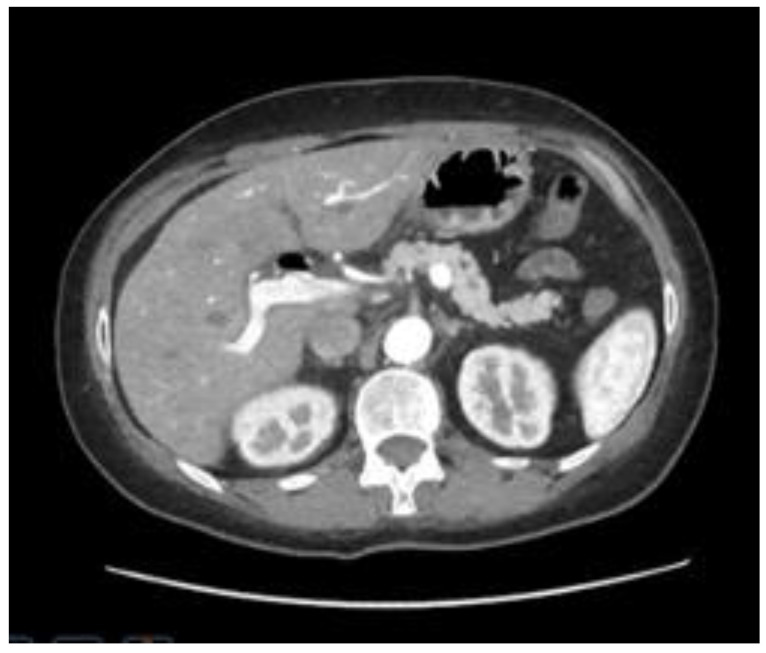
Computed tomography (CT) performed 5 days after the procedure. No gallstones were found.

Also, the voluminous gallstone was no longer detectable on a follow-up CT scan of the abdomen performed 5 days later. The patient was oral-fed again without complications and was discharged home (Figure 5).

## 3. Discussion

Diagnosis of Bouveret syndrome is based on clinical presentation in combination with imaging and/or endoscopy.

The clinical presentation includes several symptoms: nausea and vomiting in 87% of patients, abdominal pain in 71%, and hematemesis in 15% of patients (less common) [5].

Abdominal Computed tomography (CT) scan imaging is an important tool for the diagnosis of this disease, with a 100% specificity and 93% sensitivity [6]. The radiological triad of “Rigler” on the CT scan (small bowel obstruction, pneumobilia, and ectopic gallstone) is pathognomonic for this condition [7].

Endoscopy plays a central role in the management of this condition because the majority of patients with Bouveret syndrome are elderly with multiple comorbidities. Also, the rate of postoperative complications and mortality after surgery is relatively high.

Therefore, endoscopic management is emerging as the first-line therapy with various techniques: the most used is mechanical lithotripsy, which is successful in 40% of treatments. Another common method is electrohydraulic lithotripsy, which has a success rate of 21%, and less commonly, extracorporeal shockwave lithotripsy, which accounts for 4% of completely successful treatments [1]. In the case report by Makker et al. on electrohydraulic lithotripsy (EHL) [8], the authors successfully documented the treatment of an 82-year-old woman with metastatic gallbladder cancer who was unfit for surgery. However, utilizing EHL increases the risk of adverse events like bleeding and perforation because of shock wave dispersion, though water irrigation can help mitigate this risk [9]. This syndrome, historically associated with high mortality and morbidity, initially had surgery as its primary treatment. In recent years, surgery has become the secondary approach in most cases [10]. The surgical approach can be either one-stage or sequential, particularly in vulnerable patients.

The most innovative technique is fragmentation with laser lithotripsy. This minimizes tissue injury and can direct the energy precisely at the gallstone. The Holmium YAG laser is the most frequently used because it can apply high energy through a small, flexible probe [11,12,13,14,15]. In some cases, endoscopic attempts may fail. The study conducted by Crespo-Perez et al. shows that, without access to instruments like laser lithotripsy and electrohydraulic lithotripsy, they tried to remove the stone using forceps, Roth-Net baskets, Dormia baskets, and Fogarty baskets. Due to the high failure rate during these endoscopic attempts, surgical conversion was considered solely for the removal of the stone without repairing the fistula or performing a cholecystectomy [10]. After multiple fragmentation attempts, large fragments are often displaced downstream in the duodenum, potentially leading to distal gallstone ileus, at which point surgery should still be considered. Therefore, follow-ups for these patients are essential [9].

In our experience, using the laser fiber through an ERCP catheter could protect the instrument from possible damage by the laser and also stabilize the position on the gallstone to precisely control the pulse energy. The choice of the setting, in terms of the type and amount of power administration and the frequency of the holmium laser fiber, was decided based on the evidence of effectiveness shown in the literature [16].

As there is still a lack of data on the use of these devices, further studies are needed to establish which strategy to use when handling the laser.

However, laser lithotripsy may not be widely feasible because of the lack of equipment available, often supplied to endourology rooms rather than to digestive endoscopy rooms. It is important to highlight how Ong et al. showed that the combination of multiple endoscopic modalities is associated with better therapeutic success rates (*p* < 0.05) than the use of a single method [4].

## 4. Conclusions

Bouveret syndrome is a rare complication of cholelithiasis. The diagnosis is based on clinical presentation in combination with imaging and/or endoscopy. The choice certainly depends on the type of patient and the center’s availability. When available, laser lithotripsy provides better energy targeting on the stone compared to EHL or mechanical lithotripsy. In conclusion, conservative endoscopic treatment should initially be attempted, and if it fails, surgical treatment should then be performed. In fact, treating this rare condition requires a multidisciplinary approach and close collaboration not only with surgeons but also with urologists, which is essential when using the holmium laser, as in our case.

In our experience, the endoscopic approach combining laser and mechanical lithotripsy seems to be effective in fragmenting stones > 35 mm into smaller pieces, enabling their passage through the digestive lumen.

## Data Availability

The original contributions presented in this study are included in the article. Further inquiries can be directed to the corresponding author.
